# Scaling Open-Ended Survey Responses Using LLM-Paired Comparisons

**DOI:** 10.1093/poq/nfag013

**Published:** 2026-03-27

**Authors:** Matthew R DiGiuseppe, Michael E Flynn

**Affiliations:** Associate Professor, Institute of Political Science, Leiden University, Leiden, the Netherlands; Professor, Department of Political Science, Kansas State University, Manhattan, Kansas, US

## Abstract

Survey researchers rely heavily on closed-ended questions to measure latent respondent characteristics like knowledge, policy positions, emotions, ideology, and various other traits. Closed-ended questions are easy to analyze and collect, but necessarily limit the depth and variability of responses. Open-ended responses allow for greater depth and variability in responses, but are labor intensive to code. Large language models (LLMs) may help with this problem, but existing approaches to using LLMs have a number of limitations. In this paper, we propose and test a pairwise comparison method to scale open-ended survey responses on a continuous scale. The approach relies on LLMs to make pairwise comparisons of statements that identify which statement “wins” and “loses.” With this information, we employ a Bayesian Bradley-Terry model to recover a “score” on a latent dimension for each statement. This approach allows for finer discrimination between items, reduced anchoring bias, better measurement of uncertainty, and is more flexible than methods relying on Maximum Likelihood Estimation techniques. We demonstrate the utility of this approach on an open-ended question probing knowledge of interest rates in the US economy. A comparison of six LLMs of various sizes reveals that pairwise comparisons show greater consistency than zero-shot 0–10 ratings across a variety of model sizes. Further, comparison of pairwise decisions is consistent with knowledgeable crowdsourced workers.

Public opinion scholars and survey researchers are often interested in the latent traits of individual respondents, like political knowledge, literacy, comprehension, engagement, ideology, emotions, and values. Due to convenience, most scholars use closed-ended questions independently or in scales to measure these traits. However, closed-ended responses come with several undesirable properties. They introduce ceiling and floor effects. They can also introduce measurement error from false or inadvertent responses, and force an assumption of linearity on scales. Most importantly, they reduce the richness of responses and often introduce concepts that would not otherwise be apparent to respondents.

Alternatively, open-ended responses offer an unstructured and more flexible alternative that allows for in-depth and detailed responses that capture substantive uncertainty and important qualifications. However, open-ended responses have traditionally required costly human coders before they are usable in statistical analysis (Lazarsfeld 1944; Converse 1984; Geer 1991; Roberts et al. 2014; Andre et al. 2024; Haaland et al. 2024). Given a large number of potentially lengthy responses, coding the various dimensions for all respondents can be labor intensive. More often than not, this process also reduces these high-dimension data to linear discrete scales that reintroduce some issues of closed-ended questions.

Advances in text as data methods (Roberts et al. 2014) in the past 10–15 years have opened the door to automated text analysis. These techniques are particularly useful in identifying differences in sophistication or word use among groups (Kraft 2024; Zollinger 2024). However, they are ill suited for applications that require background knowledge and the comprehension of concepts embedded in long strings of text. Large language models (LLMs) excel in this regard and have the added benefit of prompting via text. Consequently, LLMs have significantly reduced the learning curve for analyzing text and the monetary cost of annotating and scaling open-ended questions (Heseltine and Clemm von Hohenberg 2024; Rathje et al. 2024; Le Mens and Gallego 2025). Notably, frontier LLMs have the added benefit of strong domain knowledge often exceeding the abilities of crowdsourced workers or undergraduate research assistants (Gilardi, Alizadeh, and Kubli 2023; Bermejo et al. 2024; Ludwig, Mullainathan, and Rambachan 2025; Ornstein, Blasingame, and Truscott 2025). While LLMs open new possibilities for survey researchers, using LLMs in place of research assistants for scaling responses typically generates unanchored scores, which also lack corresponding estimates of uncertainty. Further, LLMs vary in their output across different LLMs and even different versions of the same LLMs in classification and annotation tasks (Barrie, Palmer, and Spirling 2024). Accordingly, there remains substantial uncertainty regarding the suitability of LLMs for survey research.

In this paper, we introduce a framework for using LLMs to scale latent dimensions in open-ended responses with *pairwise comparisons* (PWC) that helps address existing concerns. First, researchers prompt an LLM to make zero-shot, independent comparisons of two random responses and indicate which is more closely aligned with the latent concept, or if they are too similar to distinguish. After collecting N comparisons, researchers use the results of the PWC to fit a Bayesian Bradley-Terry (BT) model to generate a latent variable that scales and ranks the individual respondents (Bradley and Terry 1952; Davidson 1970). The estimate of this latent dimension and the error surrounding the estimate can then be used in downstream analyses.

Just as in using PWC with human raters (Carlson and Montgomery 2017), LLM PWCs have several advantages over placement on a scale. Importantly, the approach produces estimates on a latent scale relative to other observations in the dataset rather than the unanchored responses. Next, because of the large number of comparisons possible, it is easier to recover an estimate closer to the true parameter, with smaller errors. This subsequently allows researchers to recover more nuanced differences among observations. The credible intervals around the latent variable estimates can also be used to incorporate the inherent uncertainty associated with any given observation or measure into subsequent analyses. Additionally, by forcing a binary ordering, rather than a rating, unobserved biases that do not influence rank order of pairs wash out. As we show below, this approach also allows for the generation of latent estimates even with sparse comparisons, using readily available software, reducing dependence upon customized packages for estimating pairwise comparison models.

Others have demonstrated the utility of using PWC made by crowdsourced workers to scale unidimensional latent concepts in text (Carlson and Montgomery 2017). LLMs broaden the applications of this approach. They enable pairwise comparisons on large datasets (that often exceed *N* = 1,000) that would require thousands of coder hours. Additionally, frontier LLMs have strong domain knowledge across a variety of subjects—including economics and finance—which we use in our illustration (Hultberg et al. 2024; Yang, Xu, and Qi 2024), enabling researchers to scale content that would require expert coders.

Notably, Wu et al. (2024) are the first to identify the utility of pairing LLMs with PWC in the evaluation of sentiment in “tweets” in the context of a chain of thought framework. Our contribution is to demonstrate the utility of this approach within the realm of open-ended survey responses and with zero-shot prompting and validate against a close-to-expert benchmark. We illustrate how PWC can scale “interest rate knowledge” from an original open-ended survey question about how interest rates are determined.

Our application demonstrates the usefulness of the approach and how it performs relative to zero-shot ratings. It also shows that LLMs are useful beyond sentiment analysis and multidimensional classification (Mellon et al. 2024; Le Mens and Gallego 2025). The embedded domain knowledge in LLMs can be used, in some cases, in place of close-to-expert coders. As such, it allows for scaling of concepts that are outside the reach of students or the average crowdsourced worker.

## LLMs and Pairwise Comparisons

The utility of using the embedded knowledge in LLMs to annotate, classify, and scale text has been widely demonstrated in a variety of social science fields (Gilardi, Alizadeh, and Kubli 2023; Heseltine and Clemm von Hohenberg 2024; Mellon et al. 2024; Törnberg 2025). Applications are diverse. Mellon et al. (2024) use LLMs to code the most important problem identified in open-ended responses. A number of studies use LLMs to code the sentiment or other characteristics of tweets, news reports, or other text (Gilardi, Alizadeh, and Kubli 2023; Heseltine and Clemm von Hohenberg 2024; Rathje et al. 2024; Ornstein, Blasingame, and Truscott 2025; Törnberg 2025).

These contributions demonstrate that LLMs often meet or exceed crowdsourced workers on annotation and scaling tasks. However, several well-known limitations, which also apply to human-coded data, exist. First, in scaling tasks, the responses of LLMs are unanchored. As such, what differentiates the maximum and the minimum values, or various other items on a scale, is uncertain. Second, LLMs are black boxes. There is likely unobserved bias that influences scale placement. Third, the LLM-generated responses do not produce uncertainty estimates. Consequently, subsequent models cannot discern between differences on a scale that are in fact distinct or differences that appear distinct but are in fact statistically indistinguishable. Beyond these issues, the enthusiasm for reducing the cost of dimension reduction is further dampened by concerns about replicability between LLM models and within models over time. In a series of tests, Barrie, Palmer, and Spirling (2024) show that the variance across and within models across time is “unacceptably high.”

Given these issues, can survey researchers feel confident in using LLMs to scale open-ended questions? LLMs are likely to improve and grow more consistent, and their biases may become more transparent, but until such time, researchers are faced with the task of finding alternative methods for dealing with these issues. Some of the challenges of using existing models can be improved by using LLMs to make pairwise comparisons of text and then using these judgments to generate estimates of the desired latent traits with a BT model.

Pairwise comparison is not new to social science research. They are used to measure political sophistication (Benoit, Munger, and Spirling 2019), persuasiveness of political arguments (Loewen, Rubenson, and Spirling 2012), and the dimensions of government actors (Zucco et al. 2019). The approach yields several benefits over traditional scaling techniques. First, it produces a relative assessment of each response. A well-known measurement issue with scaling text is that the responses are unanchored. This means that there is bound to be a lack of clarity on what each value on a scale means relative to the subjective construct the researcher wants to scale. Training coders can partially alleviate this concern but cannot fully resolve it. When using human coders, biases may also emerge from the order of appearance or variability across coders. It is still unclear what problem this poses for LLMs. Conceivably, prompt instructions remain constant across one-shot calls, and the actual prompt may change based on the added human written response that is unique to each respondent. As such, it is difficult to know how this impacts how the LLM places items on a scale across numerous calls. Human coders can eventually converge on an anchor by completing multiple responses assuming that they consistently apply coding rules. For LLMs, this convergence is not easily achieved. Each zero-shot or even multiple-shot call relies on a new call of the model, which entails a “clean slate” requiring a repetition of instructions. Additionally, if a long chain of thought could be achieved (at increasing cost in input tokens), some worry that LLMs exhibit recency bias (Peysakhovich and Lerer 2023). Further, efforts at multi-shot prompting rely on picking examples in an attempt to anchor scaling. Yet, it remains unclear how the choices of these examples impact the final dataset, given the opaque nature of LLMs. In sum, the subjectivity inherent in placing items on scale likely generates error that is not directly observable to the researcher.

As Carlson and Montgomery (2017) argue, PWC ameliorates this unobserved bias when using human raters. If one rater tends to rate higher or lower on a scale, this is irrelevant because the forced comparison requires a single cut point. Where on the scale an item is placed is not relevant for the final estimate unless it changes which item “wins” or “loses” a comparison. Similarly, there is concern that LLMs are inconsistent (Barrie, Palmer, and Spirling 2024), and thus a similar logic should apply. If different LLMs or differences in the prompt language (or language within a piped-in response) lead to different placement on a scale, this should only be relevant when the separate individual ratings move enough to change the outcome. For example, let us assume that a human coder or an LLM rates a knowledge response higher (lower) because grammar and spelling are flawless (sloppy). This may lead to a higher (lower) score for responses with perfect (flawed) writing. On a 0–10 scale, this might result in real differences in responses that have the same knowledge but are presented in different ways. In the PWC framework, this bias is only relevant if results flip the rank ordering, changing the winning response to a losing response.

The second benefit of the approach is that it naturally incorporates uncertainty into the estimates, allowing us to recognize when observed differences may not be statistically meaningful. While some items can be clearly distinguished from others because of real differences, in many cases, the difference between two statements is ambiguous and should be treated as such. In contrast, a simple rating-based approach may produce distinctions that are seemingly meaningful as a one-point difference on a 10-point scale—which downstream models might treat as reliable information. By explicitly incorporating uncertainty into subsequent analyses (Blackwell, Honaker, and King 2017), the risk of overinterpreting minor differences is reduced.

PWCs also make use of fine-grained and nuanced differences in a way that is difficult to implement with a fixed scale. Nuanced differences are difficult to map on a scale without placing large cognitive demands on human raters and potentially exaggerating measurement bias with LLMs (Benoit, Munger, and Spirling 2019). Consider the cognitive demands needed to determine the difference between 65 and 66 on a 100-point scale without a clear reference point or, from the perspective of a human rater, multiple cases that were coded previously. Then consider the difficulty of determining which statement is “better” than the other even if the difference is nuanced. The latter is inherently easier and quicker to assess. LLMs, while impressive, may have a similarly hard time making consistent judgments on a large scale, given that it would be difficult to have clear instructions for differences on a scale that would allow for fine-grained distinctions. By utilizing multiple pairwise comparisons, LLMs, like human coders, can produce more fine-grained differences among observations.

Until recently, PWC has relied on crowdsourced workers, students, or experts. While the benefits of PWC are clear from a measurement perspective (Carlson and Montgomery 2017), the cost of employing crowdsourced workers or RAs to engage in numerous comparisons is likely responsible for its infrequent adoption. LLMs easily remedy this concern. Scholars have demonstrated that the embedded knowledge of LLMs is sufficient to carry out these comparisons with significantly lower cost. For example, Wu et al. (2023) use LLM comparisons to recreate latent ideology scores of US senators. Di Leo et al. (2025) use a similar approach to estimate the ideology of European parties. The expertise of LLMs extends beyond political judgments. LLMs have strong knowledge of economic and psychological concepts (Geerling et al. 2023; Rathje et al. 2024).

In sum, LLMs have the embedded knowledge to replace expert coders in classification and scaling tasks. However, they are often used in a way that fails to address the subjectivity of rankings and lacks a measure of corresponding uncertainty. Below, we show that pairwise comparisons offer a better alternative to scaling approaches and can be done with reasonable costs. We also show where they may fail to produce acceptable data.

## Pairwise Comparison Workflow

**Figure 1.**
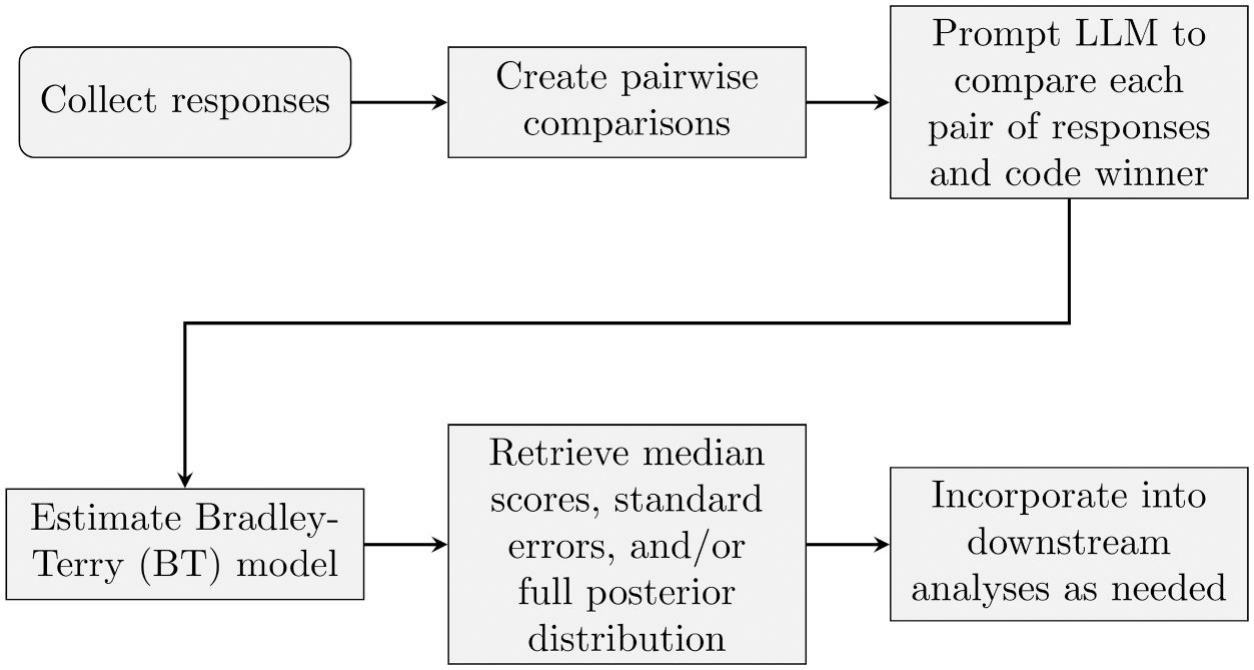
Workflow diagram of the estimation process.

Figure 1 outlines the workflow to take individual open-ended responses and create estimates to be used in subsequent analyses. After collecting open-ended survey responses, researchers first create pairs of responses. In our analysis, we started with each response and randomly paired it with 20 other responses from the dataset without replacement. Once the pairs have been assigned, researchers must develop a prompt that (1) clearly outlines the task the LLM will perform, (2) provides each of the open-ended responses, and (3) requests a response to identify which response best aligns with the concept or if they are indistinguishable. The prompt can then be used in calls to an LLM API or, for smaller models, run locally. Once the LLM returns the judgments, a BT model can be fitted to the responses.

Following the estimation of the BT model, we retrieve the median estimate and the errors. If the researcher is interested in using the estimates in downstream analyses as either an outcome or a predictor, then the researcher can sample from the posterior distribution and incorporate the sample values into subsequent analyses as needed, thereby allowing the researcher to easily incorporate the uncertainty from the BT models directly into additional models.

## MLE or Bayesian Estimation?

Traditionally BT models have been fit with Maximum Likelihood Estimation (MLE) methods. Here we adopt an alternative Bayesian framework. While others have utilized Bayesian methods for estimating similar models, the approach is not yet widespread (see Carpenter 2018; Kaye and Firth 2022; Mattos and Ramos 2022; van Paridon, Bolker, and Alday 2023). The Bayesian approach has several desirable properties relative to traditional MLE approaches. First, MLE methods can be fast, but the researcher’s ability to use MLE rests on the assumption that they have pairwise comparisons for all possible items or responses (Mattos and Ramos 2022; Kaye and Firth 2022). Where the researcher does not have pairwise comparisons for every item, MLE methods will fail to converge. Sometimes this is within the researcher’s control, but in many cases this will be out of a researcher’s reach. The cost of LLM pairwise comparison in money, time, or energy might be prohibitive. For example, in our dataset of 1,400 responses, a pairwise comparison of each response would require almost 2 million comparisons. Using 20 comparisons of each response requires only 28,000 comparisons in contrast.

Further, in cases where the number of items to be ranked is very large, MLE methods may struggle with the computational complexity. Alternatively, while Markov Chain Monte Carlo sampling methods may sometimes run more slowly, they are able to generate estimates of the desired parameters, even in cases where there are a large number of items to rank, and where not every item is paired with every other item. However, modern software for fitting Bayesian models, like Stan and its Hamiltonian Monte Carlo sampling procedures, have greatly reduced the time it takes to fit even more complex Bayesian models. Where speed might still be an issue, the choice to reduce the number of comparisons can still save the researcher time, though at the expense of larger errors in the posterior distributions of the latent parameters.

Bayesian approaches also provide us with several options for dealing with the inherent uncertainty associated with the latent estimates derived from the BT models. Packages like brms include functions like me() that can be used to account for measurement error in particular predictors. Alternatively, as we demonstrate below, researchers can simply sample from the posterior distributions of the BT estimates and run multiple iterations of downstream models to directly incorporate the uncertainty into their estimates.

Finally, while there are several R packages that can estimate BT models, many rely on MLE for estimation, or they may depend upon package maintainers to keep functions updated and working. The approach we outline here demonstrates how researchers can use Stan and brms to estimate BT models as multi-membership mixed-effects models. Depending on the structure of the pairwise comparison data, these models can be estimated using binomial or logistic regression and are generally easy to fit without relying on customized BT packages (for example, see Firth 2005; van Paridon, Bolker, and Alday 2023).

We provide a toy example in our replication materials (https://doi.org/10.7910/DVN/AXQSZB) that lays out the entire process, in R code, outlined in figure 1.

## Illustration: Scaling Economic Knowledge

To demonstrate the utility of the approach, we draw on data collected by DiGiuseppe, Garriga, and Kern (2025) that measures knowledge about monetary policy. The data, collected on the Prolific platform (*N* = 1,400) on August 6, 2024, prompted respondents with the following question: “In a few sentences and without looking it up, can you explain how interest rates (i.e., the cost of borrowing money to buy a house or car) go up or down in the US economy?” Respondents were asked to reply in 2–3 sentences. Further information about the survey data collection process can be found in Appendix A. One aim of the study was to explore how knowledge about interest rates influences individual assessments of the Federal Reserve and support for its independence. This dataset is useful for our purposes because it collects both open- and closed-ended questions relating to knowledge of the Federal Reserve, and because assessing these responses is a potentially difficult task for both human coders and LLMs. Further, the survey used quota sampling to reflect the US population in terms of age, gender, and partisanship.

We carry out this exercise with LLMs of various sizes and a mix of proprietary and open-source models (see table 1). The use of multiple models is useful for testing the limits of the approach and to compare consistency across models. These models include frontier LLMs (GPT-4o, GPT-4o mini, Llama 3.1 405B), two smaller large language models (Llama 3.1 8B and Google’s Gemma 3 4B), and a mid-sized model (Gemma 3 27B). We use API calls for the OpenAI models and the large Llama 3.1 model. We run the small and mid-sized models locally using Ollama and the R package roLlama to call on these models locally in the R environment (Gruber and Weber 2024). We used a higher-end commercial laptop, an Apple MacBook with an M3-Pro chip and 36GB of memory, for inference of these models. Other users might struggle to run the 27 Billion parameter model locally. However, these can also be accessed via API at a cost.

**Table 1.** Properties of LLMs used in illustration. The transitivity score indicates the proportion of triplets where, given A > B and B > C, the expected ranking A > C also holds. We used the following versions of the server-accessed models: GPT-4o-mini 2024-07-18, gpt-4o-2024-05-13, Llama-v3p1-405B-instruct. The Llama 3.1 405B models was accessed via Fireworks.ai API. The OpenAI models were accessed via the OpenAI API.

Model	Parameters	Open	Access	Proportion	Transitivity
		source		ties	score
Llama 3.1 405B	405 billion	Yes	API	0.024	99.06
Gemma 3 27B	27.4 billion	Yes	Local	0.057	97.42
GPT-4o mini	Unspecified	No	API	0.040	96.98
GPT-4o	Unspecified	No	API	0.008	96.12
Gemma 3 4B	4.3 billion	Yes	Local	0.021	96.04
Llama 3.1 8B	8 billion	Yes	Local	0.062	95.95

In line with the workflow we described above, we paired each response with 20 other randomly selected responses, ensuring that $\mathrm{respons}e_{i}\neq \mathrm{respons}e_{j}$. This results in approximately 30–40 total comparisons for each response, as any given respondent appears 20 times as $\mathrm{respons}e_{i}$ and anywhere from 7 to 33 times as $\mathrm{respons}e_{j}$. In total, this yields a little over 28,000 total comparisons or lines in the data. Following the workflow, we prompt each LLM with the following prompt:

You are an expert in US economic policy. Your task is to determine which of two given statements contains a more knowledgeable response to the following question: “In a few sentences and without looking it up, can you explain how interest rates (i.e. the cost of borrowing money to buy a house or car) go up or down in the US economy?” Respond with either “1” if the first statement contains more knowledge, “2” if the second statement contains more knowledge, or “0” if they are equal or incomparable. Compare these two statements and respond with “1, 2, or 0”; 1: [Statement 1], 2: [Statement 2]. Only reply with the integer 1, 2, or 0.

Table 1 shows that there are relatively few ties in most models. This suggests that the LLMs were able to distinguish between most comparisons in the dataset. We next examined the consistency of judgments across triplets, where we had judgments for A, B, and C and examined how often there was a violation of transitivity. We see that no model is perfect. Yet, the transitivity scores ([total triplets−triplets with violations]/[total triplets]) demonstrate strong consistency even among the small and mid-sized models.

Once we have the LLMs’ judgments for these 28,000+ comparisons, we estimate a Bradley-Terry model for the comparisons of each LLM. The first step is to resolve any ties in the data. Resolving these ties can be handled in several ways. Here, we randomly assign wins between the two options.^1^

Once the ties are resolved, we fit a BT model, which estimates the following:


(1) $$\mathrm{logit}\left[\mathrm{Pr}(i>j)\right]=\delta_{i}-\delta_{j}$$

The data are organized with two columns for $\mathrm{responden}t_{i}$ and $\mathrm{responden}t_{j}$, wherein the values in each vector correspond to an individual respondent ID number. A third column contains a binary indicator denoting whether $\mathrm{responden}t_{i}$ won the comparison, with a 1 indicating yes and a 0 otherwise.

Rather than using a customized package to estimate the BT model, we fit a multi-membership mixed-effects logit model using the brms package and Stan programming language (Bürkner 2017, 2018; Stan Development Team 2024; Gabry et al. 2024). The model is simply a varying intercept logistic regression model where we estimate separate intercepts for each individual respondent. Equation 2 shows the basic linear model structure to determine if player *i* beats player *j*. λ represents the varying intercept estimate for respondents *i* and *j*. The varying intercept estimates are on the log-odds scale, and are each multiplied by a weight, denoted by *W*. Because we are modeling the probability that respondent *i* wins a given comparison (i.e., Pr(i>j)), the weight for respondent *i* (i.e., $W_{i}$) takes on a value of 1, while the weight for respondent *j* (i.e., $W_{j}$) takes on a value of −1.

The outcome of Equation 2 provides us with the joint log-odds, $y_{ij}$, for a given pairing of *i* and *j*.


(2) $$y_{ij}=\lambda_{i}\times W_{i}+\lambda_{j}\times W_{j}$$

To derive the probability that player *i* beats player *j*, we simply take the log-odds value of $y_{ij}$ that we obtain from Equation 2 and plug it into Equation 3. This is simply the inverse logistic function.


(3) $$\text{Pr}(i>j)=p_{ij}=\frac{e^{y_{ij}}}{\left(1+e^{y_{ij}}\right)}$$

Equation 2 and 3 are useful because they provide a link between the regression-based syntax and the result of the BT models. Alternatively, we can work more directly with the varying intercept values for the two players and derive Pr(i>j) with Equation 4:


(4) $$\text{Pr}(i>j)=p_{ij}=\frac{e^{\lambda_{i}}}{e^{\lambda_{i}}+e^{\lambda_{j}}}$$

As we discuss above, we estimate this model as a multi-membership multilevel model using brms. The basic formula syntax of the brms model is as follows:


brms::brm(Y∼0+(1|mm(id1, id2,

weights=cbind(weight1, weight2),

scale=FALSE)

In this case, we set weight1 to equal 1 and weight2 to equal −1.

This represents the basic BT model, but we are often more interested in obtaining latent estimates of respondent knowledge than we are in the estimates of Pr(i beats j). Here we use the estimates of individual respondent knowledge provided by λ to rank-order respondents according to their knowledge and ability to accurately answer the questions posed.^2^

## Human-LLM Comparison of Comparisons

Our own prompting indicates that LLMs have a good understanding of the central process of setting interest rates. Beyond this, there is some indication that LLMs have strong domain knowledge in economics and finance (Hultberg et al. 2024; Yang, Xu, and Qi 2024). For example, an older version of ChatGPT, version 3.0, scored in the 91st percentile on the Test of Understanding in College Economics (Geerling et al. 2023). However, systematic evidence on LLMs’ domain knowledge in this, and most areas, is limited. As such, we validate the LLMs against human coders in this specific task and recommend that researchers do the same for domains where questions still remain about the alignment of LLMs with authoritative sources.

To create a human benchmark for this difficult subject, we recontacted 20 respondents from our original sample that offered an expert-level answer on our initial survey. Based on the results of the LLM ratings, we selected the top-20 responses in terms of knowledge. We then reviewed those responses to verify that they in fact provided an “expert-level” response to the question on interest rates. We then recontacted these crowdsourced workers, via the Prolific Platform, and asked them to take part in a pairwise comparison exercise to rank several 20 pairs of responses randomly drawn from our dataset.^3^ We ended up with 300 human-rated pairs. We then prompted each of the LLMs to compare these same pairs with similar instructions.

**Figure 2.**
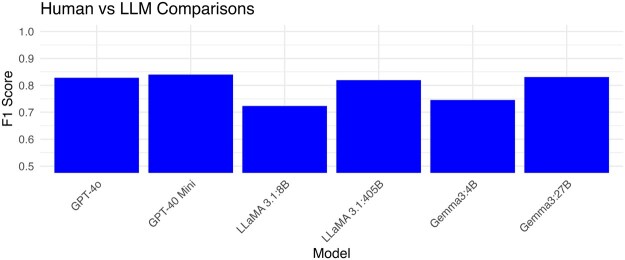
Comparisons of human vs LLM F1 scores. This figure reports the F1 scores for human-LLM comparisons of pairwise comparison results for a subset of the original dataset (*N* = 300). F1 scores indicate agreement between each LLM and human coders. Higher scores indicate greater alignment. F1 balances the model’s ability to correctly identify human-preferred responses (precision) and its coverage of human preferences (recall).

Figure 2 reports the F1 score comparing the human raters with the LLMs for the small set of responses coded by the human coders.^4^ The large frontier models (GPT-4o, GPT-4o mini, and Llama 3.1 405B) and the mid-sized model (Gemma 3 27B) exceeded an F1 score of 0.8, indicating a strong alignment with human assessments. The smaller models showed modest agreement but are clearly lagging behind the larger models. The F1 should be interpreted in context. In this exercise, we asked the human coders and the LLMs to select the profile with a “small preference” rather than indicate a tie when responses were similar. This eases interpretation and makes validation tractable. Many of the human responses are going to have similar levels of knowledge. As such, there are likely to be a high number of ambiguous cases in the dataset. Compared to classification tasks that may have clearer borders between cases, the task here will naturally lead to more disagreement. Still, we find strong F1 scores that are consistent across multiple models. This gives us confidence that the underlying task generating the data is one that is well handled by LLMs.^5^ For a more qualitative assessment, in Supplementary Material section A.4, we present several examples of comparisons and their ratings by a human and each of the LLMs.

## Comparison with Closed-Ended Responses

Thus far, we have seen that the pairwise comparison approach is well suited for large and mid-sized frontier models and the underlying task of comparisons is closely related to human decision-making. We take an additional step to compare the estimates within respondent by comparing our BT estimates of individual respondents to their own responses on closed-ended questions in the same survey. We compare these responses with the BT estimates derived from the pairwise comparisons of the largest open-source model, Llama 3.1, with 405 billion parameters. We chose this model given our preference for an open-source model motivated by concerns of replicability.

**Figure 3.**
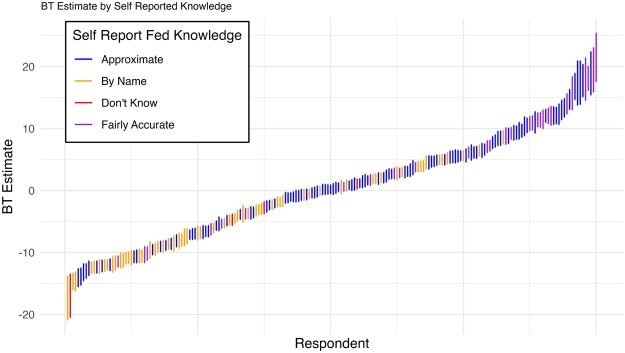
BT estimates by self-reported knowledge of the Fed. Here we randomly selected 200 responses (for visibility) and plot the BT estimates in order of knowledge. The 95 percent confidence intervals (bars) of the estimates are colored based on responses to a query about a respondent’s self-reported knowledge of the Federal Reserve.

**Figure 4.**
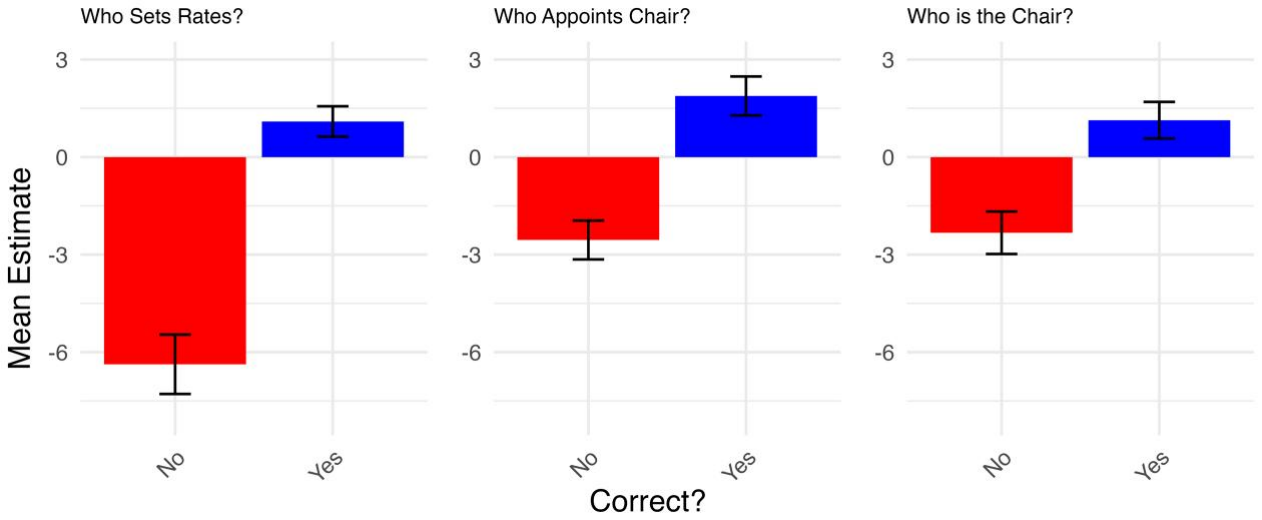
Mean BT estimate by correct and incorrect answers to factual questions about the Fed.

In figure 3, we plot the BT estimates in order but classify them based on how a respondent responded to the question “How familiar are you with the following US institution: The Federal Reserve.” We see, in line with expectations, that those that “have a fairly accurate idea of the duties of the institution” and “have an approximate idea of the duties of the institution” score higher on the scale. Those that “only know the institution by name” or “don’t know” the institution rank consistently lower on the scale. Next, we turn to factual questions about the Federal Reserve. Given the large role of the Federal Reserve in setting interest rates, knowledge about the institutional structure of the Federal Reserve should be strongly related to knowledge about interest rates. Figure 4 presents the mean BT estimate by correctness of three factual questions in which respondents had to pick the correct answer from four choices. We see that those who could not identify which institution in the US government sets interest rates, who appoints the chair, and those who could not identify the Fed chair have significantly lower BT estimates. This gives us confidence that the BT estimates align with the underlying construct—knowledge about the Federal Reserve and interest rates.

## Comparing LLMs

**Figure 5.**
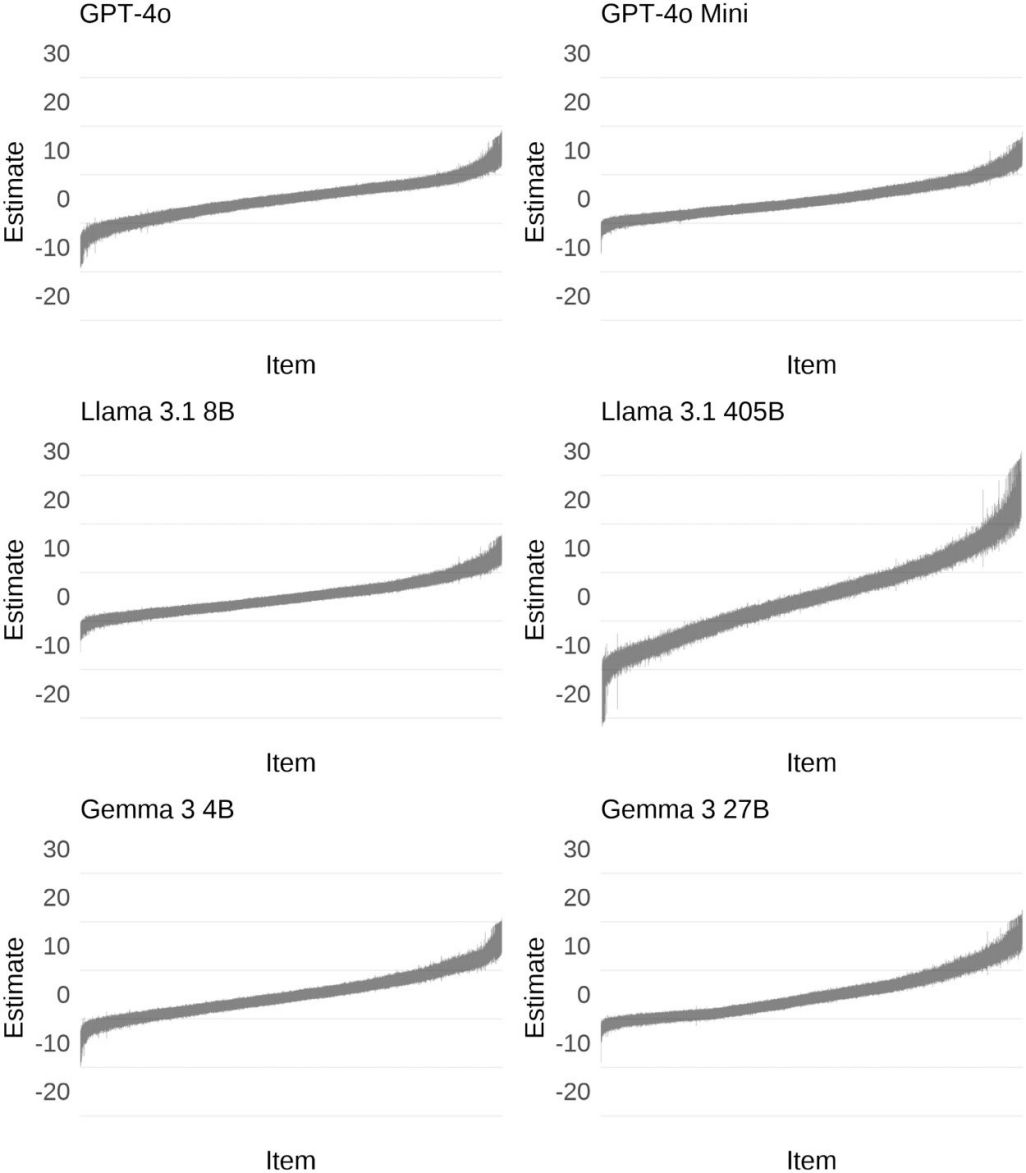
Bayesian Bradley-Terry estimates of interest rate knowledge by LLM. Each panel plots the 95 percent credible interval for the posterior distributions for each respondent in our dataset. Responses (Items) are sorted highest to lowest within each panel.

We now proceed to compare the final BT estimates from the pairwise comparisons of each LLM. First, figure 5 plots the 95 percent credible intervals around each respondent’s latent estimate from each of the six models. In each figure, we sort latent estimates according to the median of their posterior distributions from the lowest to highest respondent knowledge ability within each model. The plots demonstrate a similar pattern across all but the smallest model.

Table 2 presents the correlations of the final BT scores for each model. Each correlation exceeds 0.87. Correlation among the largest models (GPT-4o and Llama 3.1 405B) exceeds 0.95. This suggests that, with this specific task, the LLMs are largely in agreement about what constitutes a highly knowledgeable answer. This provides confidence that LLMs can produce consistent scales when used to produce pairwise comparisons.

**Table 2.** Correlation of BT estimates of pairwise comparisons by LLM.

	Gemma 3	Gemma 3	Llama 3.1	Llama 3.1	GPT
	4B	27B	8B	405B	4o mini
Gemma 3:27B	0.894	—	—	—	—
Llama 3.1:8B	0.908	0.884	—	—	—
Llama 3.1:405B	0.898	0.942	0.902	—	—
GPT-4o Mini	0.899	0.931	0.908	0.949	—
GPT-4o	0.876	0.919	0.879	0.948	0.926

For comparison, we also prompted each LLM to engage in zero-shot numerical ratings of individual statements. We asked each LLM to place each statement on a scale reflecting the knowledge about interest rates from completely incorrect or irrelevant (0) to highly knowledgeable and accurate (10).^6^

**Figure 6.**
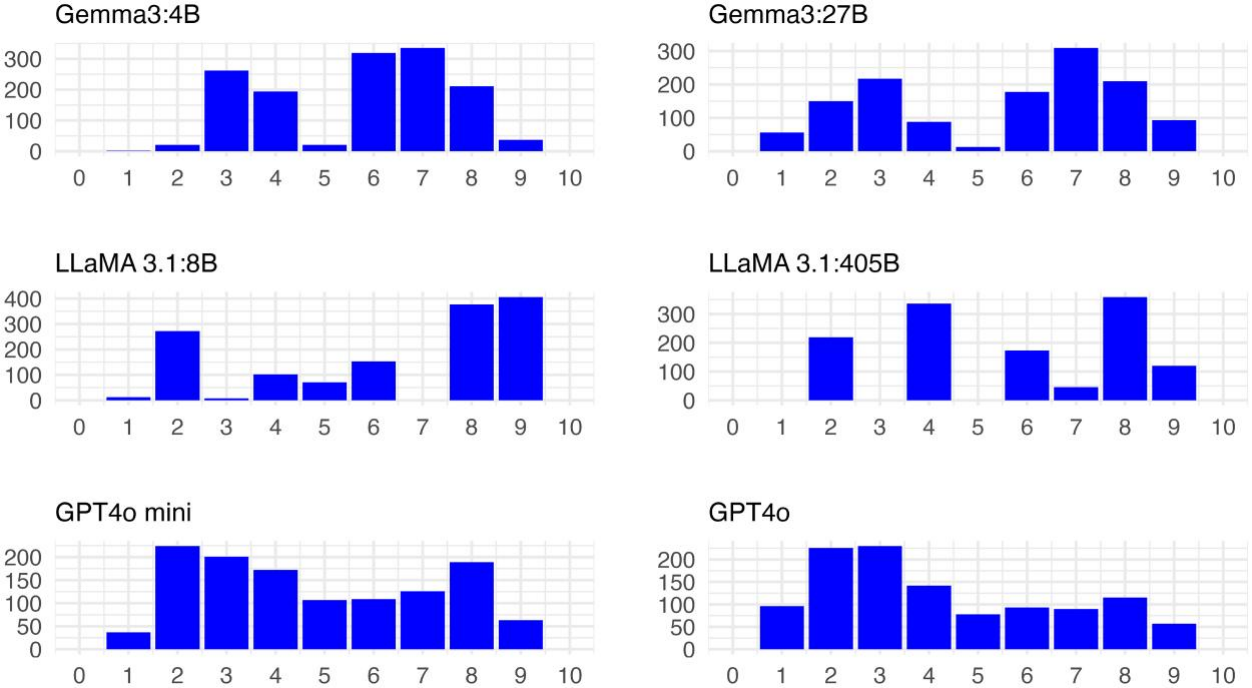
Distribution of ratings. This figure presents the distributions of the 0–10 ratings of interest rate knowledge for each of the LLMs (*N* = 1,402).

Figure 6 plots the distribution of these results, and table 3 presents the correlations of these ratings. Several things stand out. First, we see that while the models are given a range of values to place a statement, several models rely on a fraction of those values. As such, zero-shot ratings may inherently limit the nuance of their output and thus miss key distinctions between responses. This also gives further pause, as it appears that the patterns do not follow an apparent logic. It suggests that there is an inherent bias in selecting some numbers over others. Next, we see that the correlation among the models is strong. Yet, the consistency across models falls short of the consistency of the BT estimates. For example, among the two largest models (GPT-4o and Llama 3.1 405B), the ratings are correlated at 0.85, compared with 0.95 in the BT estimates.

**Table 3.** Correlation of 0–10, one-shot ratings by LLM.

	Gemma 3	Gemma 3	Llama 3.1	Llama 3.1	GPT
	4B	27B	8B	405B	4o mini
Gemma 3:27B	0.841	—	—	—	—
Llama 3.1:8B	0.775	0.785	—	—	—
Llama 3.1:405B	0.824	0.914	0.807	—	—
GPT-4o mini	0.822	0.879	0.751	0.911	—
GPT-4o	0.792	0.862	0.724	0.847	0.882

While our analysis here is limited to one domain, the findings suggest that one-shot ratings may be a less costly option, though they underperform compared to a pairwise comparison approach. The findings of an additional illustration, which we present in Supplementary Material section A.2, support this conclusion.

The last step in our workflow is to draw from the distribution of Bradley-Terry estimates and use a multiple (over)imputation framework to recover an aggregate estimate that incorporates the uncertainty in the latent variable. Here we use our latent variable, interest rate knowledge, as a predictor of support for central bank independence.^7^ We expect that people with greater knowledge will favor independence. As such, we estimate linear models that include interest rate knowledge plus potential confounders: education and income.

**Figure 7.**
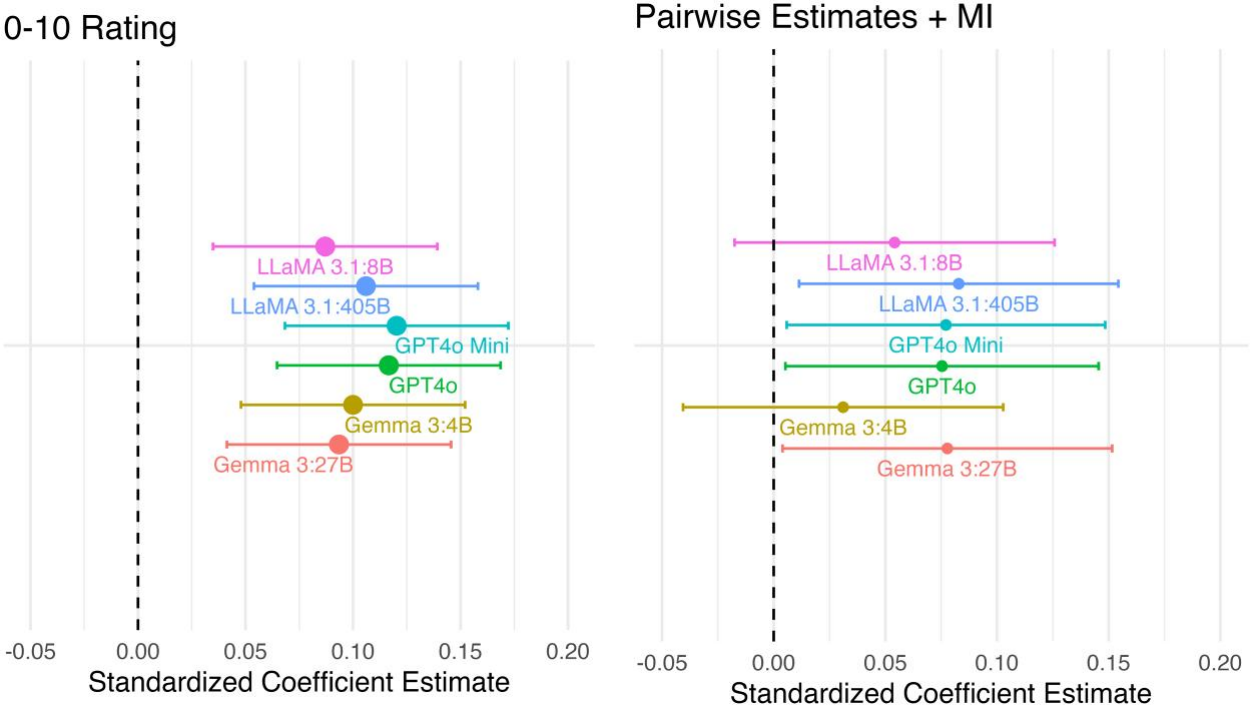
Coefficient of interest rate knowledge on support for central bank independence. Both panels plot the standardized coefficients and 95 percent CI of “interest rate knowledge” in a linear model predicting support for central bank independence for each of the LLMs used in our analyses. Each model also includes controls for income and education. The left plot relies on a 0–10 one-shot rating on knowledge.

The right panel of figure 7 presents the standardized coefficients of interest rate knowledge for each the LLM-derived latent variables and the corresponding 95 percent confidence interval. We see that the point estimates for the larger models appear to be bigger. If there is indeed a relationship, it suggests that the more capable models are more adept at picking up the underlying concept. For comparison, we also plot the standardized coefficients of similar linear models for the zero-shot 0–10 ratings in the left panel. First, the two approaches provide point estimates of different sizes. The coefficients of the BT estimates are about 75 percent the size of the rating coefficients. Further, the confidence intervals around the BT estimate coefficient are considerably larger, given the incorporation of the estimate uncertainty.^8^ Depending on the underlying LLM comparisons, this can result in the difference between a significant or insignificant result. Overall, the analysis illustrates the potential consequences of relying solely on one-shot numerical ratings in downstream models.

## Scope and Limitations

Open-ended questions have been underutilized because of the cost of hand-coding text or the limitations of current algorithms to uncover latent dimensions that researchers care about. LLMs have opened the door to using open-ended questions more often and in more applications. We go further and suggest that LLMs also unlock the benefits of PWC to scale open-ended responses. If hand-rating text is expensive, a sufficient number of comparisons in large datasets is, in most applications, prohibitive. LLMs can again reduce barriers to this scaling technique that hold several theoretical advantages over one-shot ratings. Notably, PWC reduces bias, increases precision, and allows for measures of uncertainty. Further, we show that, at least when it comes to this task, LLMs’ judgments correspond closely to expert or near-expert human coders, there is high agreement among different LLMs, and they outperform numerical ratings.

Beyond our application here, PWC by LLM can be deployed in numerous ways. For example, researchers can pull multiple dimensions from the same source. Open-ended text can hold multiple dimensions, and often those concepts may confound each other. The process we outlined above can be used to scale numerous dimensions independently and thus “control” for confounding dimensions in downstream analyses. Tapping different dimensions from the same text might also be helpful in experimental settings where researchers may wonder if they have manipulated the intended variable or a closely related concept. Similarly, our approach could be helpful in the choice of survey instruments in pilot analysis, as researchers now can test if different question wordings tap the similar constructs by pooling responses and looking for separation in the BT estimates.

The method itself can also be refined to reduce costs. For example, researchers can implement an adaptive BT model to reduce the absolute number of comparisons (Maystre and Grossglauser 2017) by focusing more attention on statements that are close in proximity.

While the method is useful, it does have several limitations. First, researchers must be able to identify and phrase a question that will reveal the latent dimension of interest. Some concepts may still be better probed in the context of discrete factual questions, while others might benefit from a longer exposition. Further, the domain knowledge of LLMs has not been fully mapped. It is still necessary to validate the use of LLMs with high-quality benchmarks (Gilardi, Alizadeh, and Kubli 2023).

Second, some concepts may be easier to identify than others. The task is best suited to applications where researchers require a single, easily inferred, dimension. We carried out an additional illustration (see Supplementary Material section A.2) in which we asked an LLM to identify “uncertainty” in respondents’ expectations of the economic consequences of government action. Here, we see less agreement across LLMs. However, the method still produces more consistent output than numerical ratings. Consequently, researchers should do their due diligence to demonstrate that LLM output is consistent across and within models, and that the findings are not dependent on the judgments of a single LLM.

Third, the ability of various LLMs to accurately and consistently evaluate the factual content of respondents’ answers depends on the integrity of the underlying LLM model and training data. If a given LLM cannot “retain” a piece of factual information, then its ability to evaluate how factual a given response is will likely not be stable over time (Khatun and Brown 2024). Other factors, like the framing of questions/prompts or the inclusion of extra or unnecessary language in prompts, can produce incorrect responses. Similarly, while multiple LLMs may rank particular options as the most likely correct responses, it is difficult to assess the degree of confidence or uncertainty across various LLMs, or how stable they are over time (Wang et al. 2024).

Finally, while LLMs may potentially bring new life to the use of open-ended questions in survey research, they also bring risks beyond the structural and mechanical problems of the LLMs themselves. The increasingly widespread use of LLMs for completing various user tasks may interfere with efforts to collect user knowledge from surveys. We were suspicious that several of the open-ended responses from our Prolific respondents were themselves generated by LLMs. To explore this further, when we recontacted respondents to serve as our human benchmarks, we again asked them to answer the question about interest rates. This time, we hid an additional request in the html in small transparent font. We asked that the response to “mention Alan Greenspan.” This served the purpose of providing a very specific instruction that is unlikely to be included without prompting, but also one that would not arouse too much suspicion if read by the human pasting into the survey. We found that five of our top 20 respondents were using AI to answer the open-ended question. We removed these responses from the final dataset. While the use of LLMs by survey respondents was not detrimental to our analysis, it does show that respondents may rely on LLMs for cognitively demanding tasks like open-ended questions. The prospect of AI agents completing surveys on their own raises the risk that entire survey forms will be completed by AI. Consequently, survey researchers, whether using open or closed questions, should design strategies to identify these responses and drop them from the dataset.

## Supplementary Material

nfag013_Supplementary_Data

## Data Availability

Replication data and documentation are available at https://doi.org/10.7910/DVN/AXQSZB.

## Footnotes

1 We randomly assign a winner to help illustrate the simplest implementation of the procedure. We discuss alternative approaches to handling ties in Supplementary Material section A.6.

2 When estimating the models we use broadly regularizing priors to facilitate model convergence.

3 As we note below, we screened out those that have used LLMs themselves.

4 The F1 score reports a balance between the model’s precision (correctness of its positive predictions) and recall (ability to identify all positive instances), representing a combined measure of the model’s accuracy on the positive class. $F1\;\text{Score}=2\times \left(\frac{\text{Precision}\times \text{Recall}}{\;\text{Precision}+\text{Recall}}\right)$. $\text{Recall}=\frac{\text{True}\;\text{Positives}}{\text{True}\;\text{Positives}+\text{False}\;\text{Negatives}}$. $\text{Precision}=\frac{\text{True}\;\text{Positives}}{\text{True}\;\text{Positives}+\text{False}\;\text{Positives}}$.

5 We also compared our human raters to a chain-of-thought prompt and a reasoning model (OpenAI’s o4-mini). Neither model demonstrated an improvement over the large frontier models employed in our analysis.

6 The prompt reads as follows: “You are an expert in US economic policy. Your task is to rate the given statement on a scale of 0–10 based on how knowledgeable it is in response to the following question: In a few sentences and without looking it up, can you explain how interest rates (i.e. the cost of borrowing money to buy a house or car) go up or down in the US economy? Rate the following statement on a scale of 0–10, where 0 is completely incorrect or irrelevant, and 10 is highly knowledgeable and accurate: [statement]. Respond ONLY with a single integer from 0 to 10, with no additional text.”

7 The respondents were asked, “People disagree about the independence of the Federal Reserve. Do you think this independence should be decreased or increased?” after reading a vignette that describes the current independence of the Federal Reserve.

8 Note that increasing comparisons will likely shrink the confidence intervals.

## Appendix A. AAPOR-Required Disclosure Elements

**First Data Source:** Survey data collected by DiGiuseppe, Garriga, and Kern (2025) via the Prolific online platform to measure knowledge about monetary policy and interest rates. The data used in the example in the Supplementary Material was collected by DiGiuseppe and Shea (2025) via the Prolific online platform to measure uncertainty about the consequences of a debt ceiling increase.

**Data Collection Strategy:** Online survey employing both closed- and open-ended questions to capture respondent knowledge about how interest rates are determined in the US economy.

**Research Sponsor and Conductor:** DiGiuseppe would like to acknowledge the support of the European Research Council (ERC) Starting Grant #852334

**Measurement Tools/Instruments:** Interest rate illustration: “In a few sentences and without looking it up, can you explain how interest rates (i.e., the cost of borrowing money to buy a house or car) go up or down in the US economy?” Respondents were asked to reply in 2–3 sentences.

**Uncertainty Illustration:** “In one or two sentences, what do you think will happen if the government DOES NOT increase the debt ceiling?” Experimental manipulation included images and accompanying text designed to increase uncertainty about debt ceiling consequences.

Additional closed-ended questions measured self-reported knowledge of the Federal Reserve and factual knowledge questions about Federal Reserve structure and leadership: “Eight times a year a group of people in a US institution meet to review data and set the US’s basic interest rate level. Which US institution sets base interest rates?” (The Federal Reserve, The US Treasury, The Congress, The Consumer Finance Protection Bureau, The Internal Revenue Service); “How familiar are you with the following US institution: The Federal Reserve” (I don’t know the institution, I only know the institution by name, I have an approximate idea of the duties of the institution, I have a fairly accurate idea of the duties of the institution); “Who appoints the chair of the Federal Reserve?” (The President, The Senate, The House of Representatives, The Treasury, The Federal Reserve Board); “Who is the current Chair of the US Federal Reserve?” (Jamie Dimon, Warren Buffett, Jerome Powell, Janet Yellen, Joseph Biden).

**Population Under Study:** US adults. The study used quota sampling to reflect the US population in terms of age, gender, and partisanship.

**Methods Used to Generate and Recruit the Sample:** Nonprobability sample recruited through the Prolific platform. Quota sampling methodology used to achieve balance on age, gender, and partisanship. No specific eligibility requirements mentioned beyond standard Prolific participation criteria. Geographic location: United States.

**Method(s) and Mode(s) of Data Collection:** Web-based survey administered through the Prolific platform. Language: English.

**Dates of Data Collection:** Interest Rate Survey: August 6, 2024; Uncertainty Survey: May 2nd, 2023.

**Sample Sizes and Precision of Results:** Initial Interest rate sample: *N* = 1,400; Initial Uncertainty sample: *N* = 1,486; Human validation subsample: *N* = 20 expert-level respondents recontacted for pairwise comparison validation.

**Whether and How the Data Were Weighted:** Quota sampling was employed to reflect US population demographics on age, gender, and partisanship. No poststratification weighting procedures are described.

**How the Data Were Processed and Procedures to Ensure Data Quality:** Responses were analyzed using six different large language models (LLMs): GPT-4o, GPT-4o Mini, Llama 3.1 405B, Llama 3.1 8B, Gemma 3 4B, and Gemma 3 27B. Pairwise comparison methodology used where each response was randomly paired with 20 other responses. Human validation conducted with expert-level respondents. Detection method for AI-generated responses: hidden HTML request to “mention Alan Greenspan” identified 5 of 20 validation respondents using LLMs, who were removed from analysis. Bayesian Bradley-Terry models estimated using Stan and brms packages in R.

**Dispositions or Response or Participation Rates:** Not provided in the manuscript.

**Sample Sizes:** Unweighted sample size of *N* = 1,400 for main analysis. Human validation subsample of *N* = 20 (reduced to *N* = 15 after removing AI-generated responses).

**Measurement and Model Specification:** Bayesian Bradley-Terry models estimated using multimembership mixed effects logistic regression. Model formula: Y ∼ 0 + (1|mm(id1, id2, weights = cbind(weight1, weight2), scale = FALSE)). Software: R packages brms and Stan. Each response paired with 20 others, resulting in approximately 28,000 total comparisons. Note that the “weight1” and “weight2” entries in the model formula simply reflect the +1 and −1 weights used to create the equations described in Equations 1–4 in the main manuscript.

**A General Statement Acknowledging Limitations of the Design and Data Collection:** This study uses a nonprobability convenience sample from Prolific with quota sampling, which may limit generalizability to the broader US population. The detection of AI-generated responses among validation participants highlights the challenge of ensuring authentic human responses in online surveys. The LLM analysis methodology is novel and requires validation across different domains and tasks.
