# Supplementary material for: Scaling Open-Ended Survey Responses Using LLM-Paired Comparisons
Source: Public Opin Q. 2026 Mar 27;90(3):630–56. doi: 10.1093/poq/nfag013 (PMC13287519; doi:10.1093/poq/nfag013)
Supplement: nfag013_Supplementary_Data [file nfag013_supplementary_data.pdf]

Supplementary Appendix for Scaling Open-ended Survey Responses Using LLM-Paired Comparisons

Matthew DiGiuseppe
Associate Professor
Leiden University
mdigiuseppe@gmail.com*

Michael Flynn
Professor
Kansas State University
meflynn@ksu.edu

September 22, 2025

*Corresponding author.

Contents

A Additional Analyses and Information **2**

A.1 Chain of Thought Prompting & Reasoning Models 2

A.2 Additional Illustration: Uncertainty 4

A.3 Incorporating Character Length of Responses 8

A.4 Response and Decision Examples 14

A.5 A note on ELO 17

A.6 Resolving Ties 18

A.7 LLM Model Details 19

A Additional Analyses and Information

A.1 Chain of Thought Prompting & Reasoning Models

In addition to asking an LLM to return just a final answer on which statement best aligned with the latent dimension of interest (knowledge), we also attempted a Chain of Thought (CoT) pair-wise comparison prompting approach following the recommendation of (Wu et al., 2023) and used a reasoning model (GPT-4o mini) to carry out our pair wise tasks. For the reasoning model, we simply provided the same prompt we used in the comparisons analysis we presented in the main manuscript. For the CoT analysis, we used the following prompt with the Llama 3.1 405b model:

"You are an expert in US economic policy. Your task is to determine which of two given statements contains a more knowledgeable response to the following question:", "In a few sentences and without looking it up, can you explain how interest rates (i.e., the cost of borrowing money to buy a house or car) go up or down in the US economy?", "Follow these steps to complete the task:", "Step 1: Write out your evaluation of Statement 1, discussing its strengths, weaknesses, and gaps in knowledge.", "Step 2: Write out your evaluation of Statement 2, discussing its strengths, weaknesses, and gaps in knowledge.", "Step 3: Compare your evaluations of the two statements and explain which one demonstrates greater knowledge, or why they are equal or incomparable.", "Step 4: Based on your reasoning, provide your final decision.", "Your response should include the full reasoning for each step, and the final decision must be presented as:", "Final Decision: [1] or Final Decision: [2] or Final Decision: [0]", "Here are the statements to evaluate:", "1:", [statement1], "2:", [statement2], "Write out your full evaluation and conclude with the final decision in the specified format."

We find, contrary to our expectations, that the CoT prompt performed worse than the direct prompt against the "close to expert" benchmark. This builds on recent evidence that CoT prompting may have been useful for smaller and older models but is no longer necessary

with frontier LLMs (Meincke et al., 2025).

Similarly, the reasoning model did not generate appreciable gains over the non-reasoning models. As such, we present only the results from the more cost-effective one-shot, non-reasoning models in our main analysis. We suspect that for this task, the frontier LLMs are sufficient and there are no appreciable gains to be made by further prompting either internally (reasoning models) or externally (CoT).

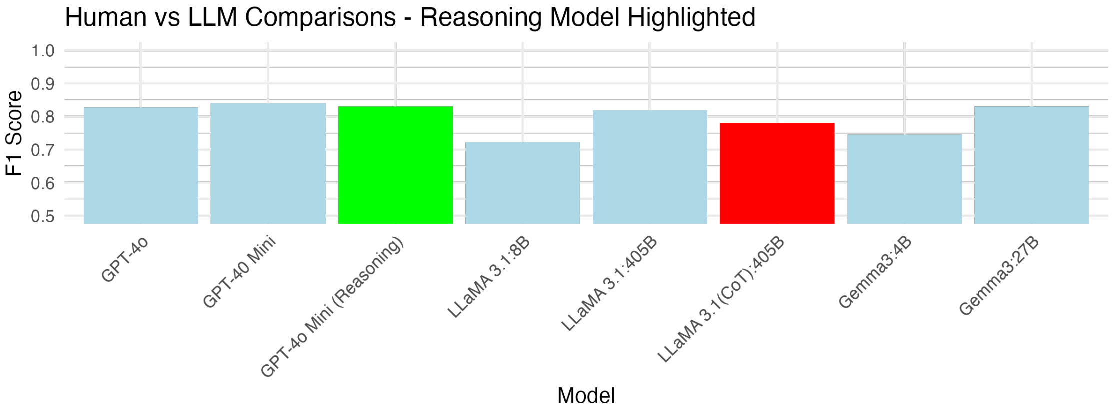

Figure A1: F1 - LLM-Human Comparison including Chain of Thought prompt

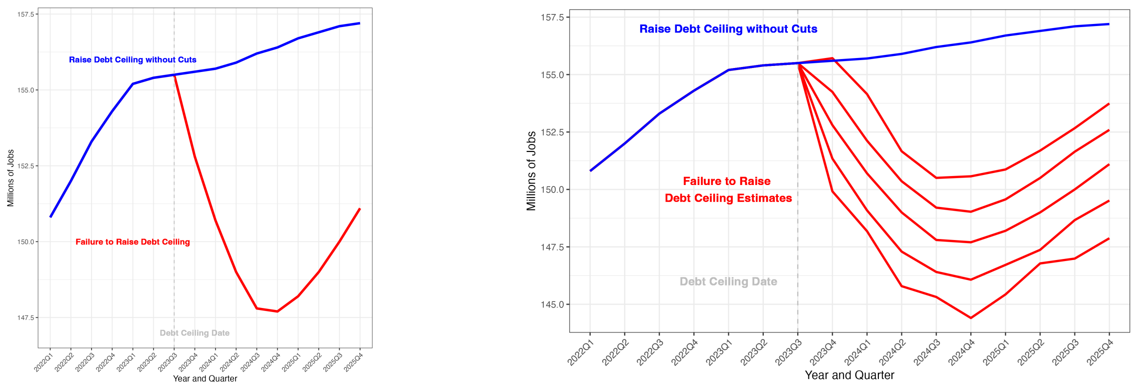

(a) Control Figure

(b) Treatment Figure

Figure A2: Caption place holder

A.2 Additional Illustration: Uncertainty

In addition to the illustration above, we also applied our framework to an experimental setting where the variable of interest is a dependent variable. In a recent paper, DiGiuseppe and Shea (2025) attempt to manipulate respondents' uncertainty over the consequences of a debt ceiling breach in the run-up to the 2023 debt ceiling deadline in the United States. The survey was deployed on a quota representative sample on Prolific (N=1486). Figure A2 presents the images in the experiment that accompanied text that reinforced the goal of the figures. As a manipulation check, the authors asked respondents, post treatment, "In one or two sentences, what do you think will happen if the government DOES NOT increase the debt ceiling?" In their analysis (reported in the Appendix), the authors used and LLM (GPT-4) to rate the uncertainty of respondent expectations in each response on 5, 11, or 101 point scale. Using this measure, they find that the LM ranked the statements in the 'uncertain' condition as having higher and statistically significant uncertainty score when using each of these scales.

Here, we apply a pairwise comparison approach to this data and try to replicate their findings and examine differences between different models and between the BT estimates and 0-10 ratings as we did with the example in our main analysis.

The figures and tables in this section indicate a few things. First, there is much less

consistency in the model output in this task. The correlations are much lower among both the BT estimates and the 0-10 rankings. The BT estimates appear to be more consistent among the high-end models. Among the 3 frontier models (Llama 3.1 405b, GPT-4o and GPT-4o mini), the correlation of the final output ranges from 0.59 to 0.82. Still, this may be too low to have confidence in any particular model for this task. Unfortunatley, we do not have a human-benchmark to compare these findings. Lastly, the figure A5 shows that LLM choice can have a dramatic impact on inference where LLMs are inconsistent in their comparisons.

The exercise suggests that the usefulness of LLMs to scale latent variables from text is conditional on the specific task. Still, it is worth noting that, the pairwise comparisons still demonstrate more consistency than the zero-shot ratings. Theoretically, this aligns with the benefits of pairwise comparisons to eliminate bias that does not impact the ranking of two statements.

	Gemma 3:4B	Gemma 3:27B	Llama 3.1:8B	Llama 3.1:405B	GPT4o Mini	GPT4o
Gemma 3:4B	1.00	0.35	0.69	0.21	0.49	-0.02
Gemma 3:27B	0.35	1.00	0.23	0.50	0.47	0.58
Llama 3.1:8B	0.69	0.23	1.00	0.34	0.55	0.03
Llama 3.1:405B	0.21	0.50	0.34	1.00	0.79	0.82
GPT4o Mini	0.49	0.47	0.55	0.79	1.00	0.64
GPT4o	-0.02	0.58	0.03	0.82	0.64	1.00

Table A1: **Correlation of BT Estimates of Pairwise Comparisons by LLM: Uncertainty**

	Gemma 3:4B	Gemma 3:27B	Llama 3.1:8B	Llama 3.1:405B	GPT4o Mini	GPT4o
Gemma 3:4B	1.00	0.25	0.27	0.28	0.19	0.31
Gemma 3:27B	0.25	1.00	0.53	0.58	0.69	0.60
Llama 3.1:8B	0.27	0.53	1.00	0.59	0.52	0.45
Llama 3.1:405B	0.28	0.58	0.59	1.00	0.64	0.59
GPT4o Mini	0.19	0.69	0.52	0.64	1.00	0.57
GPT4o	0.31	0.60	0.45	0.59	0.57	1.00

Table A2: **Correlation of Uncertainty Ratings by LLM**

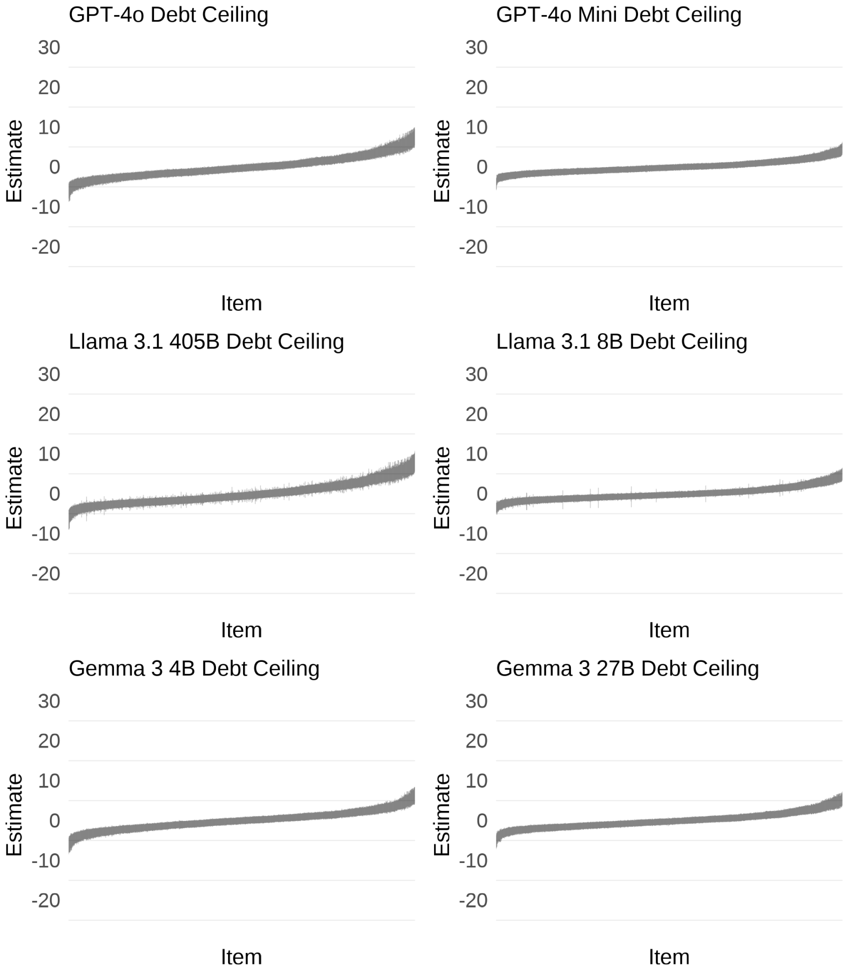

Figure A3: Bayesian BT knowledge scores

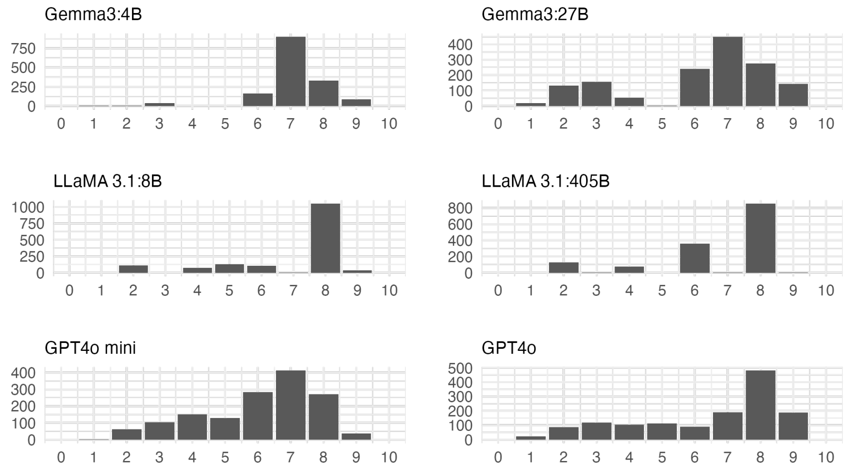

Figure A4: Distribution of Uncertainty 0-10 Ratings by LLM

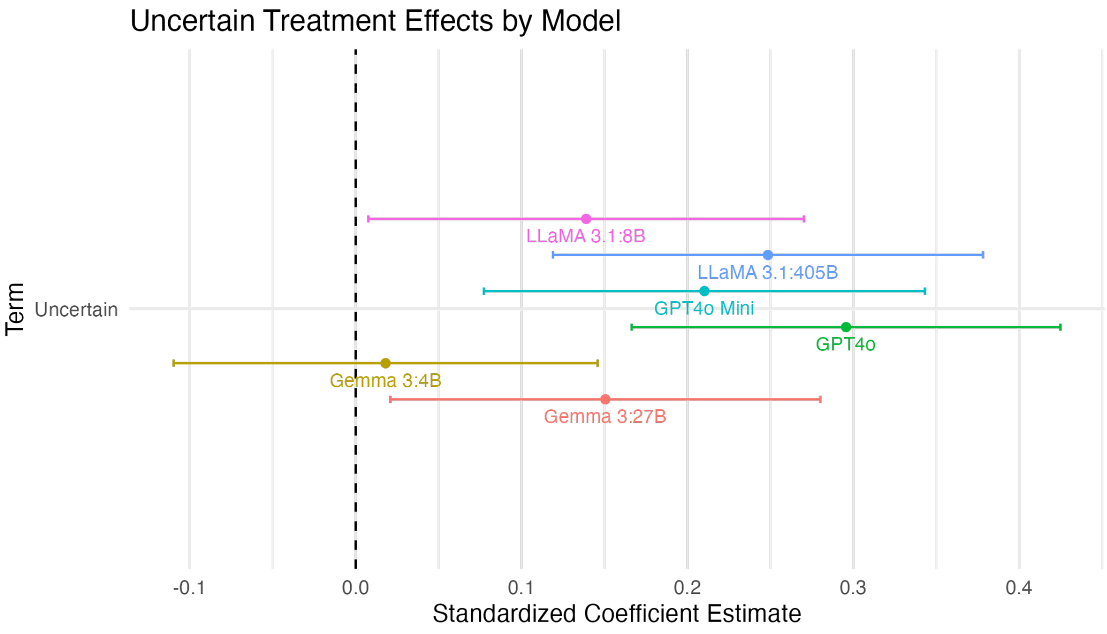

Figure A5: **ATE of treatments based on LLM codings of 'Uncertainty DV':** The point estimates indicate the average treatment effect and bars indicate the 95% confidence intervals.

A.3 Incorporating Character Length of Responses

It is possible that LLMs are evaluating the knowledge contained in responses by standards other than the factual content of the answer. For example, it may be that LLMs are inclined to more highly rate longer responses over short responses. In such cases the risk would be that responses with longer answers are more likely to win a given matchup when we make our paired comparisons.

To better understand this issue we run our six primary LLM models again using our Bradley–Terry framework, but this time we include a variable that adjusts for the difference in the length of the responses as measured by the number of characters contained in each response. In these models we measure the difference in response lengths as follows:

$$\text{Difference}_{ij} = \text{Response Length}_i - \text{Response Length}_j \tag{1}$$

Where Response Length is simply a count of the number of characters in the respondent's answer. Positive values indicate that respondent i had a longer answer and negative values indicate that respondent j had a longer answer. We use this difference-based measure because using the length of a single player's response tells us little without the additional context of the other player's response length. With this measure we should expect positive values to correlate positively with a win for player i, assuming the LLM is privileging longer answers.

Figure A6 shows the distribution of the response lengths across all individual respondents, as well as the distribution of the differences in responses that we use in the Bradley–Terry models to adjust for the relative length of the players' responses. The bulk of the distribution of the responses is largely concentrated around 150 characters, with a mean of 161 and a median of 129, with some outliers with several hundred characters. However, after randomly pairing respondents we can see that the distribution fo the difference variable is normally distributed, with the bulk of the respondents having fairly small differences.

Figure A7 shows the coefficients for the differences in response lengths. Across all six

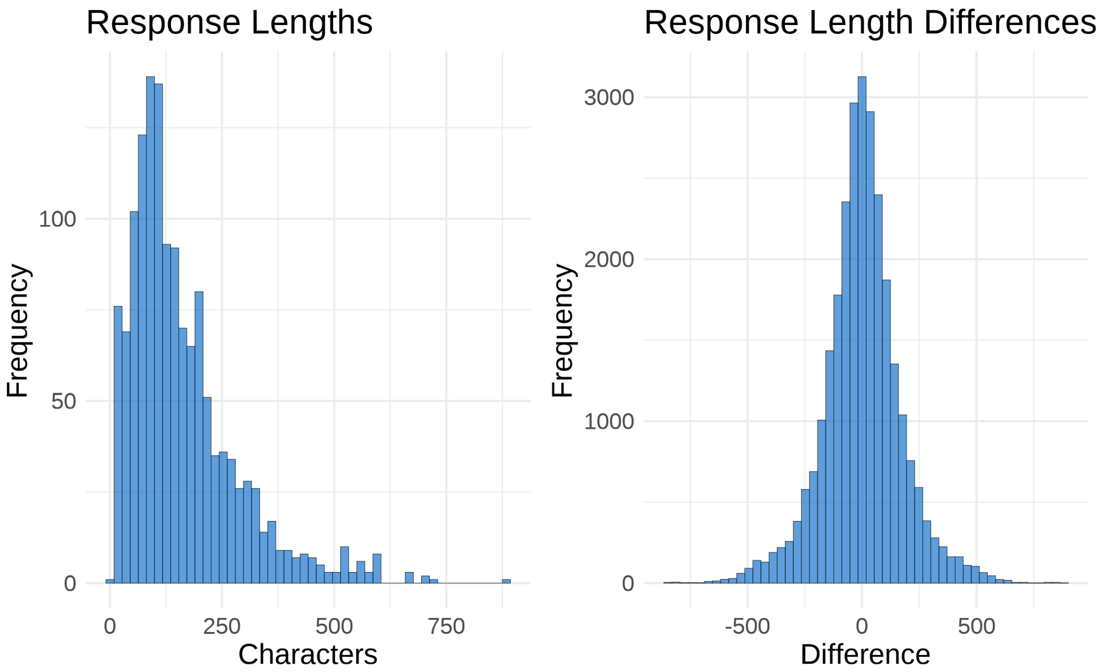

Figure A6: In this model, we compare BT estimates that include a parameter for character length with the models we estimated above that omitted character length.

of the models we examine we find positive coefficients for the response length difference variable, indicating that as the length of player i's response increases compared to player j, there is an increase in probability that the LLM chooses player i as the winner. If the length of the response made no difference then we should expect to see coefficients and posterior distributions clustering around 0. In this case the coefficients and posterior distributions for all six models fall well above 0, indicating a positive effect for response length relative to a player's opponent.

Additionally, the magnitude of these coefficients is fairly small at first glance, with most values concentrated around a value of 0.02 and the Llama 3 405b model producing a slightly larger coefficient of approximately 0.045. Though it is normally difficult to tell the substantive magnitude of the effects from looking at a logit coefficients on their own, the population intercept is set to 0 in our models, meaning that this coefficient represents the population-average effect for a one-unit change in the response length difference variable relative to 0. Furthermore, the median positive and negative difference values are approximately 91,

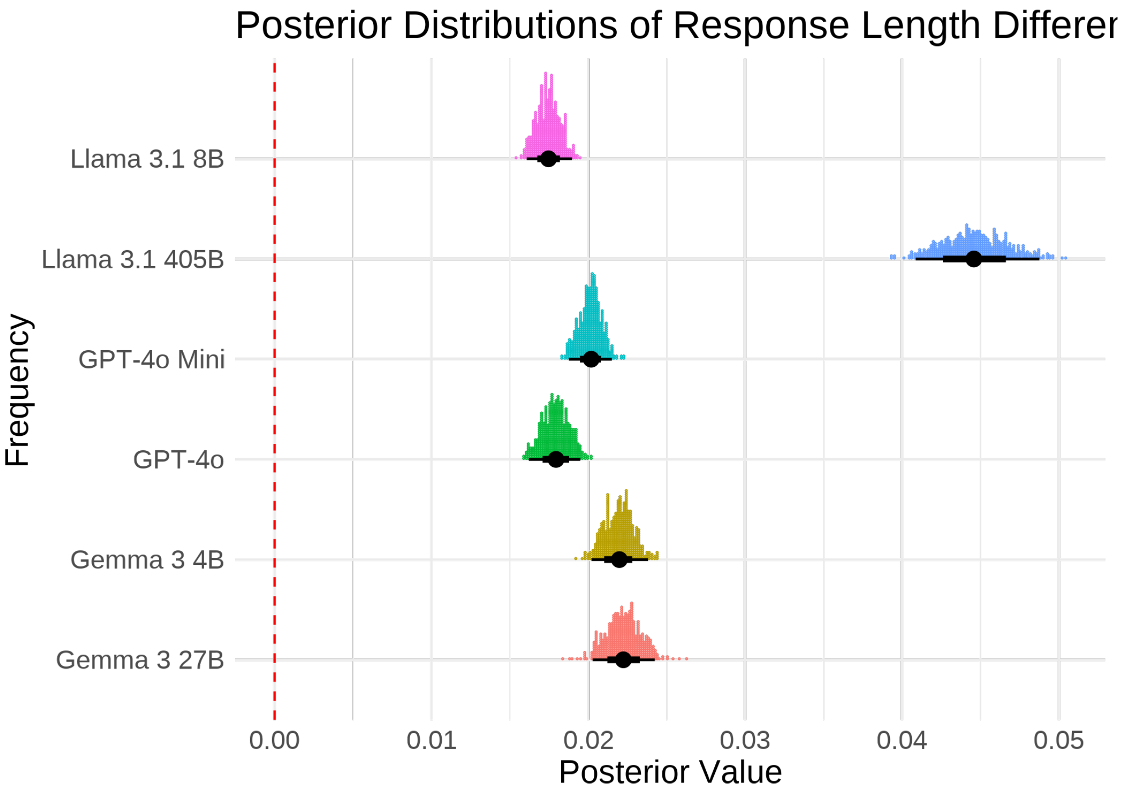

Figure A7: Coefficients for the variable measuring the difference in response lengths for players i and j in the Bradley–Terry models.

which means that this coefficient is better understood in practice as representing a change of approximately ± 1.8–2.0 on the log odds scale in many practical cases. This is a fairly sizable shift given the practicable range of the log odds scale.

As a further check we look at the correlation between the individual-level knowledge estimates from our base models and those generated by the models that include the covariate for the difference in response lengths. Figure A8 shows the correlation between all of the base models and the those with the response length variable. Ultimately we find that both sets of models are producing knowledge estimates that are largely in line with one another. The lowest correlation coefficient is 0.88 with a median of 0.91 and a mean of 0.93. In general the models are both producing knowledge estimates and rankings that are comparable with only minor slippage between the base models and the adjusted models.

Finally, we include two additional figures to show how the rankings of randomly chosen

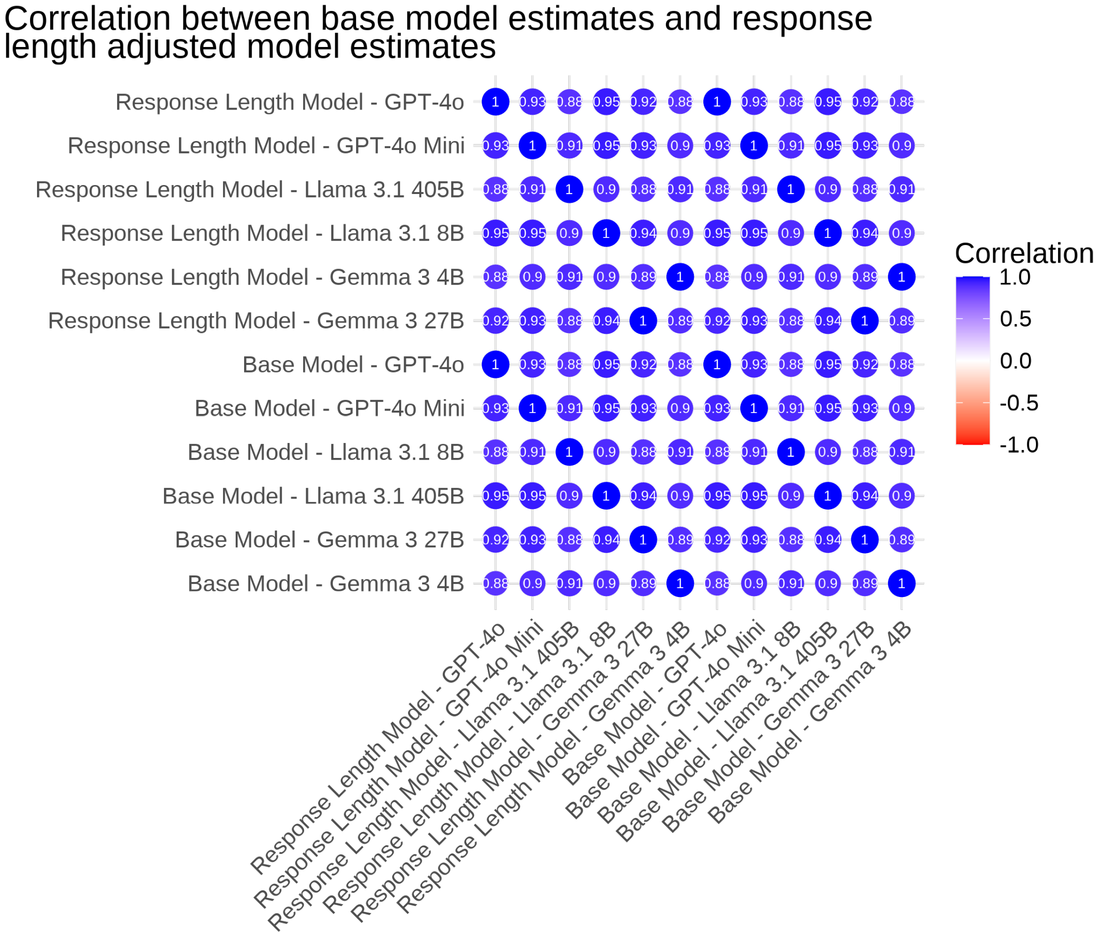

Figure A8: Correlation plot for the individual player rankings generated from the base models including in the main text and the supplementary models that include a variable adjusting for the difference in the length of the respondents' answers.

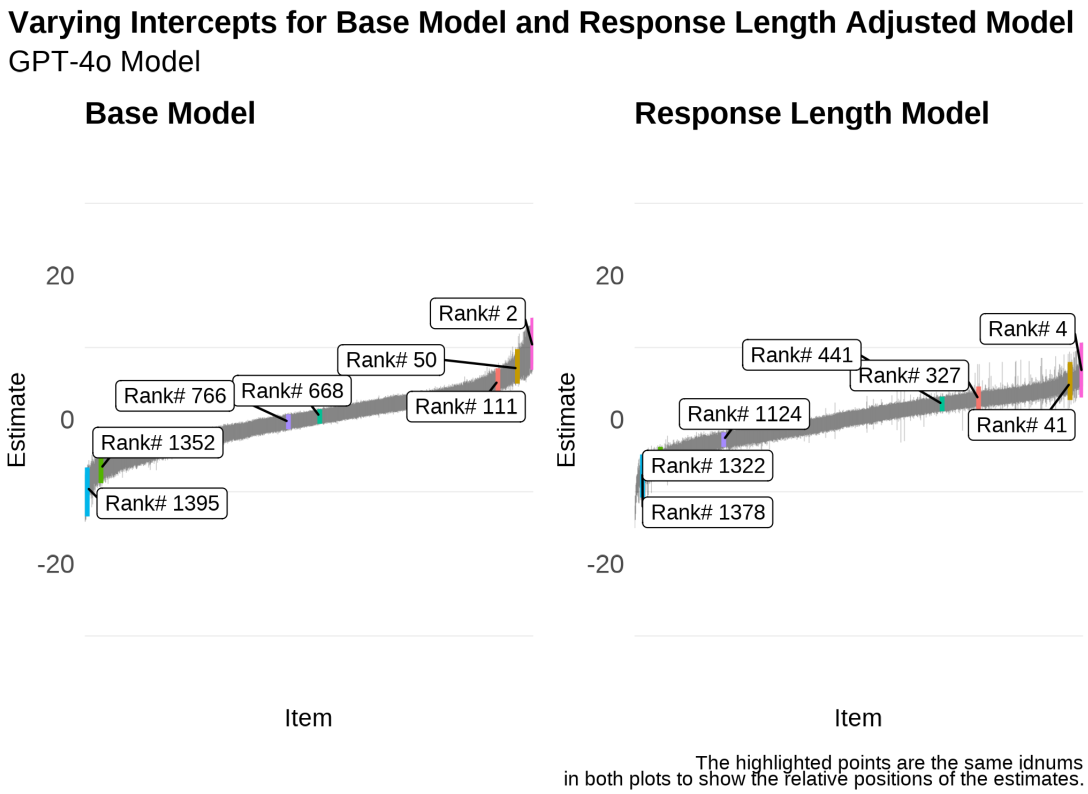

Figure A9: Varying intercept plots for the GPT-4o base model showing rankings for randomly selected ID numbers.

respondent IDs compare between the base model and the adjusted model. Figure A9 shows the rankings for seven randomly chosen respondents based on the baseline BT models and the models that adjust for the differences in the length of the responses. For the most part we see consistency in terms of the relative ordering of the chosen respondents, but we do see some shifting along the x axis indicating that there is some shifting in the absolute rankings of the respondents across models. In general these shifts are fairly modest, though some across see larger swings than others. For example, in Figure A9 we see that the light green bar moves from the 668th highest rank to the 327th. The remaining respondents are fairly consistent in their general ranks between the base and adjusted models.

GPT-4o is one of the more common LLMs and the coefficient for the adjustment variable is in line with all of the other LLMs with the exception of the Llama 405b model. The coefficient for this model was by far the largest, suggesting that rankings may be more

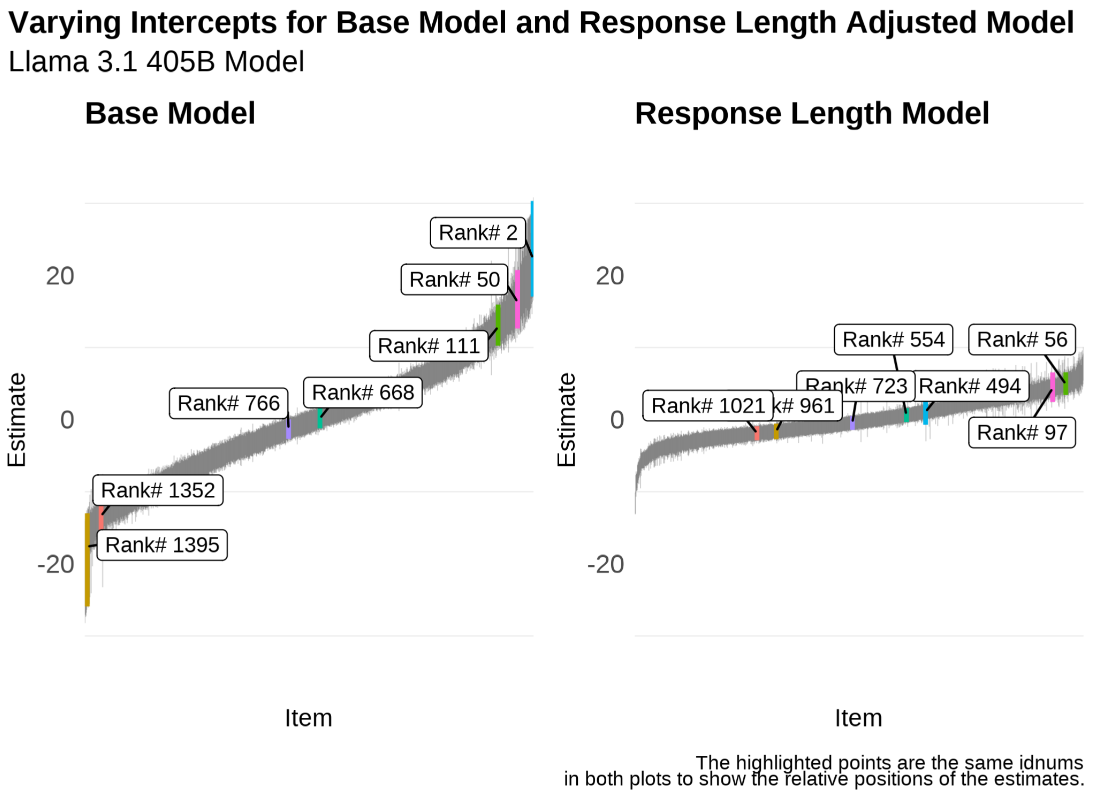

Figure A10: Varying intercept plots for the Llama 405b base model showing rankings for randomly selected ID numbers.

sensitive to the inclusion of the response length adjustment variable.

As above, Figure A10 shows the rankings of the same randomly selected respondents in both the base and adjusted models. Here we see more substantial shifting for some of the respondents. For example, we see that the light blue bar slips from 2 to 494. Alternatively, the salmon bar moves from 1,352 up to 1,021 in the adjusted models. Others, like the pink bar, remain fairly consistent, ranking at 50 and 97.

On the whole these shifts appear to be fairly mild and both sets of models still appear to capture useful variation in rankings across the full range of respondents. Parsing differences between individual respondents is, of course, a much more difficult task, and one benefit of the Bayesian approach is that the posterior distributions around each individual varying intercept estimate remind us of the uncertainty inherent in this work, while also providing us with a way to incorporate that uncertainty into downstream analyses.

A.4 Response and Decision Examples

Table A3: Comparison for Pair 1: Statements 834 vs 1323

**Statement A (ID: 834)**	**Statement B (ID: 1323)**
If the Fed(Federal Reserve) raises the interest rates it is a snowball effect amongst lenders with credit cards and small loan companies causing the most damage to the average person. Those companies will begin charging close to 30% on what you borrow from them. In the long run, a 1% increase in a 30 year mortgage could also turn into hundreds of thousands of extra dollars that the person with the mortgage has to pay.	Generally the Fed sets their interest rates based on what they want the economy to do. Other interest rates, for banks, credit cards, consumer loans, etc., are keyed in some way to the federal loan rate. Also plain supply and demand can influence interest rates: too many people chasing too little available money can drive up interest rates, and the reverse can happen.

**Rater**	**Preference**
Human	Statement B
GPT-4o	Statement B
GPT-4o Mini	Statement B
Llama 3.1 8b	Statement B
Llama 3.1 405b	Statement B
Gemma 3 4b	Statement B
Gemma 3 27b	Statement B

Table A4: Comparison for Pair 2: Statements 461 vs 1373

**Statement A (ID: 461)**	**Statement B (ID: 1373)**
I think that interest rates are high right now so trying to purchase something is hard because you cant purchase as much of anything because of interest rates	The Fed looks at how the economy is performing and raises the interest rates or lowers them in order to avoid inflation or a recession.

**Rater**	**Preference**
Human	Statement B
GPT-4o	Statement B
GPT-4o Mini	Statement B
Llama 3.1 8b	Statement B
Llama 3.1 405b	Statement B
Gemma 3 4b	Statement B
Gemma 3 27b	Statement B

Table A5: Comparison for Pair 3: Statements 1295 vs 779

**Statement A (ID: 1295)**	**Statement B (ID: 779)**
Im not sure. I know the federal reserve decides whether to raise, lower, or keep the same interest rates. I think they raise them when things are going well economically and lower them when prices go up on consumer goods.	Interest rates are set by the Federal Reserve. The try to control inflation rates and economic stimulation by raising rates for the former and lower rates for the later.

**Rater**	**Preference**
Human	Statement B
GPT-4o	Statement B
GPT-4o Mini	Statement B
Llama 3.1 8b	Statement B
Llama 3.1 405b	Statement B
Gemma 3 4b	Statement B
Gemma 3 27b	Statement B

Table A6: Comparison for Pair 4: Statements 154 vs 1020

**Statement A (ID: 154)**	**Statement B (ID: 1020)**
I dont know.	Supply and demand has an impact. If demand goes down, rates will too.

**Rater**	**Preference**
Human	Statement B
GPT-4o	Statement B
GPT-4o Mini	Statement B
Llama 3.1 8b	Statement B
Llama 3.1 405b	Statement B
Gemma 3 4b	Statement B
Gemma 3 27b	Statement B

Table A7: Comparison for Pair 5: Statements 1373 vs 827

**Statement A (ID: 1373)**	**Statement B (ID: 827)**
The Fed looks at how the economy is performing and raises the interest rates or lowers them in order to avoid inflation or a recession.	When inflation goes up the interest rates go up to slow down the spending.

**Rater**	**Preference**
Human	Statement A
GPT-4o	Statement A
GPT-4o Mini	Statement A
Llama 3.1 8b	Statement B
Llama 3.1 405b	Statement A
Gemma 3 4b	Statement B
Gemma 3 27b	Statement A

Table A8: Comparison for Pair 1: Statements 162 vs 755

**Statement A (ID: 162)**	**Statement B (ID: 755)**
I dont know. Supply and demand	I dont know but I assume it has to do with the state of the economy and value of the US dollar and stock markets. Everything is plummeting and getting worse so in turn interest rates soar.

**Rater**	**Preference**
Human	Statement A
GPT-4o	Statement B
GPT-4o Mini	Statement B
Llama 3.1 8b	Statement B
Llama 3.1 405b	Statement B
Gemma 3 4b	Statement B
Gemma 3 27b	Statement A

A.5 A note on ELO

Alternative methods of rating players and predicting the probability of wins, like Elo-based approaches, are similar to the approach used here and are workable in other contexts but make less sense for this kind of application.

First, these approaches generally assume scores are updated as players face new opponents in a sequential fashion. In these cases the content of each "match" for each player is different. In our case the content for each player remains the same across matches. Nor are games sequential in our case. While we could engineer sequential "games" in the data, such an iterative process would substantially increase the computational intensity of the estimation process for no clear gains in terms of estimation accuracy.

Additionally, Elo methods require users to specify a *K-factor* parameter, which specifies the maximum possible adjustment to an individual player's rating resulting from a win or loss, which again assumes sequential matches between players. The approach we adopt here requires fewer assumptions on the part of the user (See Berg (2020)).

A.6 Resolving Ties

In our framework, we randomly assign a winner to help illustrate the simplest implementation of the procedure and one that does not introduce bias but may introduce noise. Given the relatively small number of ties in our data we can do so without significantly impacting the results. However, we could easily modify the approach we use here to use an ordered logit model in place of the binary logit. In this case outcome variables could be coded as ordered factor variables with values of "Player i loses", "Tie", and "Player i wins". This approach comes at the expense of added computational intensity with some models taking at least twice as long to run as the binary logit models, but with negligible returns. For example, the correlation between the estimates of the binary logit and ordered logit for the GPT-4o models is 0.99. Alternatively, users can also prompt an LLM to resolve the ties for them before estimating the models (for more see Davidson, 1970).

A.7 LLM Model Details

Table A9: LLM checkpoints and quantisation details used in the study

Model	Parameters	Quantisation	File size (GB)	OLlama digest
Gemma-3 4B	4.3B	$Q4_{KM}$	3.3	a2af6cc3
Gemma-3 27B	27.4B	$Q4_{KM}$	17.4	a418f583
Llama-3.1 8B	8.0B	$Q4_{KM}$	4.9	46e0c10c
Llama-3.1 405B (Fireworks)	405B	server-side bf16	n/a	fireworks.ai
GPT-4o-mini 2024-07-18	unspecified	server-side bf16	n/a	OpenAI API
GPT-4o 2024-05-13	unspecified	server-side bf16	n/a	OpenAI API

References

Berg, Arthur (2020, July). Statistical Analysis of the Elo Rating System in Chess. *CHANCE 33*(3), 31–38. https://doi.org/10.1080/09332480.2020.1820249.

Davidson, Roger R (1970). On extending the bradley-terry model to accommodate ties in paired comparison experiments. *Journal of the American Statistical Association 65*(329), 317–328. https://doi.org/10.1080/01621459.1970.10481082.

DiGiuseppe, Matthew and Patrick E Shea (2025). Information, uncertainty, and public support for brinkmanship during the 2023 debt limit negotiations. *British Journal of Political Science 55*, e14. https://doi.org/10.1017/S0007123424000462.

Meincke, Lennart , Ethan Mollick, Lilach Mollick, and Dan Shapiro (2025). Prompting science report 2: The decreasing value of chain of thought in prompting. Technical report, Generative AI Labs, The Wharton School of Business, University of Pennsylvania. SSRN Working Paper. https://doi.org/10.48550/arXiv.2506.07142.

Wu, Patrick Y , Jonathan Nagler, Joshua A Tucker, and Solomon Messing (2023). Concept-guided chain-of-thought prompting for pairwise comparison scaling of texts with large language models. *arXiv preprint arXiv:2310.12049*. https://doi.org/10.1109/BigData62323.2024.10825235.
